# Solitary Keratoacanthoma of the Scalp: A Case Report

**DOI:** 10.7759/cureus.51176

**Published:** 2023-12-27

**Authors:** Norah A Alghamdi, Mashael A Taj, Shuaa A Alamri, Jamal A Taj, Shahad J Alshowaikhat, Tariq Jaber, Galia Jadkarim

**Affiliations:** 1 College of Medicine and Surgery, Batterjee Medical College, Jeddah, SAU; 2 Department of Medicine, King Faisal Hospital, Makkah, SAU; 3 Obstetrics and Gynecology, King Fahad Armed Hospital, Jeddah, SAU; 4 Orthopedic Surgery, College of Medicine, King Saud Bin Abdulaziz University for Health Sciences, Jeddah, SAU; 5 Bariatric, Minimal Invasive, and General Surgery, National Guard Health Affairs, Jeddah, SAU; 6 Breast Oncoplasty, Endocrine, and General Surgery, National Guard Health Affairs, Jeddah, SAU

**Keywords:** squamous cell carcinoma (scc), excisional biopsy, keratoacanthoma, solitary, scalp

## Abstract

Keratoacanthoma (KA) is a pruritic, rapidly growing cutaneous neoplasm originating from the infundibulum of the hair follicle on the sun-exposed area. Usually presents as a dome-shape with a centralized keratinous plug. Whether KA is benign or malignant is challenging due to its similarity to squamous cell carcinoma (SCC). In this case study, we present a 57-year-old patient who came to the surgery clinic with a rapidly growing ellipse-shaped nodule on her scalp for two years, which was diagnosed and treated as solitary KA by excisional biopsy. This case represents the first reported KA on the scalp of an elderly patient in Saudi Arabia.

## Introduction

Keratoacanthoma (KA) is a rapidly growing, pruritic, cutaneous neoplasm, typically dome-shaped measuring 1-2 cm, originating from the hair follicle [1,2]. Pathologists commonly classify KAs as "well-differentiated squamous cell carcinoma, keratoacanthoma variant," as about 6% of KAs can progress to SCC if untreated [3]. Cutaneous squamous cell carcinomas (cSCC) are keratinocyte carcinomas, originating from the keratinocytes located in the epidermis or adnexal structures [4]. Differentiating between KA and SCC is challenging due to their similarities and the lack of reliable diagnostic criteria [5]. It occurs between ages 45 and 69 with a male-to-female ratio of 2:1. It affects sun-exposed skin, mainly the face and the dorsum of the upper extremities. While most KAs originate from hair follicles, rare cases on mucous membranes suggest an origin from surface epithelium. Factors such as UV radiation exposure, male gender, advanced age, fair skin, smoking, and alcohol consumption contribute to KA development [6]. KA onsets manifest as a rapidly growing nodule over four to five weeks, followed by four to eight weeks of stability, naturally regresses, and eventual resolution over six months to two years, often leaving a depressed scar due to keratin expulsion [7]. Various subtypes exist including solitary, subungual, mucosal, giant, centrifugum marginatum, Grzybowski’s generalized eruptive, and Ferguson-Smith syndrome multiple KAs [8]. Treatment of KA, crucial due to its link to SCC, often involves excision with 4 mm margins or electrodesiccation and curettage for smaller lesions. Larger or aggressive tumors might require tissue-sparing options like Mohs micrographic surgery, especially in cosmetically sensitive areas or cases with perineural invasion. Non-surgical methods, such as topical creams (e.g., 5% imiquimod or 5% 5 fluorouracil (5-FU)) and intralesional treatments (e.g., methotrexate or 5-FU), have shown promise [9-12]. We report here a case of scalp KA that was surgically treated in a 57-year-old female patient. The resected site on the scalp healed well without complications and no recurrence.

## Case presentation

**Figure 1:**
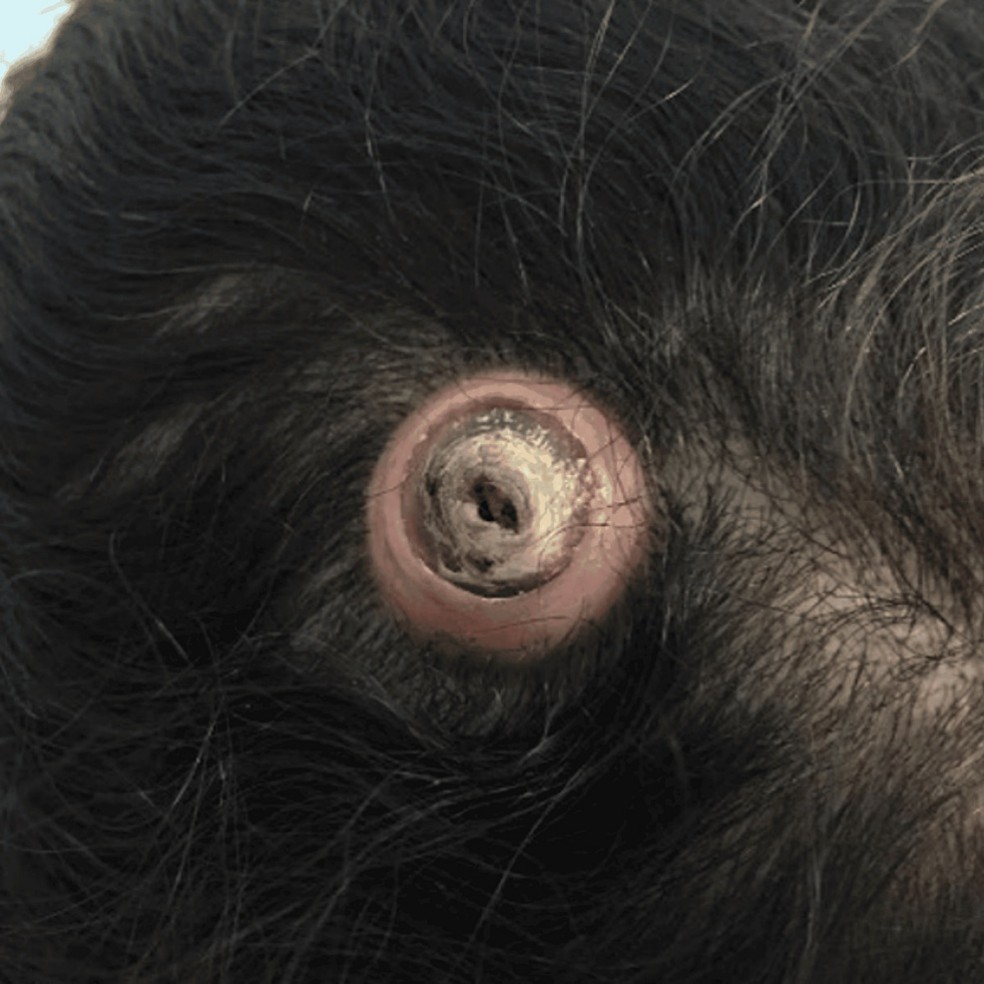
Rapidly growing ellipse-shaped nodule on the scalp

A 57-year-old Saudi female presented to the surgery clinic, with a rapidly growing ellipse-shaped nodule on her scalp for two years (Figure 1). The mass did not regress with time and was filled with serous fluid. The patient had a medical history of diabetes and hypertension, and her family history was not significant. Physical examination revealed an oval-shaped mass with a central scar, no discharge, no fluctuation, and painless. The patient was booked for a minor operation for an excisional biopsy, where an elliptical incision was made. The mass was excised with a grossly negative margin, and the wound was closed by primary closure. The postoperative pathology report showed a dilated pore of the winner and no evidence of malignancy. The gross pathological description includes a skin ellipse measuring 1.5 × 1.2 × 0.4 cm with a raised skin lesion and a central crater measuring 1.1 cm. Microscopically, sections showed skin-abundant pilosebaceous follicles with dilated infundibulum containing lamellated keratin and lined by stratified squamous epithelium with peripheral basal cells and no cytologic atypia. At the one-year follow-up, the patient's pathology results confirmed free margins and no malignancy, requiring no further intervention. The wound healing was smooth, and no recurrence.

## Discussion

KA is a low-grade, rapidly growing, dome-shaped skin tumor typically ranging from 1-2 cm and characterized by a centralized keratinous plug [9]. KA predominantly occurs between the ages of 45 and 69, with a higher incidence in males, affecting primarily sun-exposed areas [6]. Differentiating between KA and SCC poses challenges due to their similarities and the lack of reliable diagnostic criteria [3]. Fortunately, KA’s distinct morphological characteristics and growth patterns usually allow for its diagnosis in most cases. KA tends to be both exophytic and endophytic, displaying a central keratin-filled crater. In contrast, cutaneous SCCs are mainly endophytic, often presenting with ulceration. Histopathologically, KA exhibits specific characteristics. While the superficial epithelium at the tumor's edge seems normal, a noticeable acute angle is observed at the central crater's rim. Keratin fills the crater, and the epithelial cells at its base proliferate downward, often triggering a significant chronic inflammatory reaction. Dyskeratosis, similar to well-differentiated SCC, is evident, either in individual cell keratinization or the formation of keratin pearls [13]. The progression of KA involves three clinical stages. Proliferative stage: marked by a rapidly enlarging papular nodule. Mature stage: characterized by a bud- or hemisphere-shaped tumor containing a central keratinous core. Involutional or resolving stage: the tumor becomes keratotic and necrotic, ultimately leading to a puckered, hypopigmented scar as it resolves [6]. Our patient presents with a scalp mass spanning more than a year. She had diabetes and hypertension, raising the possibility of immunosenescence. Surgical excision remains the treatment of choice in the majority of KA cases. Although KAs generally regress, predicting the final size and growth timeframe is challenging, potentially leading to disfiguring lesions in the interim. Our patient underwent surgical excision with primary closure, the primary treatment option for most cases. However, various other treatment methods have been documented, including systemic retinoids, intralesional and topical 5-FU, intralesional methotrexate (MTX), laser therapy, electrodesiccation and curettage, radiotherapy, and photodynamic therapy [13]. She presented to the dermatology for follow-up. The lesion showed good healing without complications or recurrence, but unfortunately, her hair did not grow after surgery. Other differential diagnoses of a lesion consistent with KA include SCC, amelanotic melanoma, molluscum contagiosum, prurigo nodularis, metastatic lesion to the skin, Merkel cell carcinoma, nodular basal cell carcinoma, ulcerative basal cell carcinoma, nodular Kaposi sarcoma, hypertrophic lichen planus, deep fungal infection, atypical mycobacterial infection, foreign body reaction, and verruca vulgaris [6].

## Conclusions

KA is a benign neoplasm with unique clinical features but histopathological features similar to cutaneous SCC. We report the first case of solitary KA on the scalp of an elderly Saudi woman who was successfully treated by surgical excision.
